# Diminishing returns of disease-modifying treatment in older participants of multiple sclerosis clinical trials

**DOI:** 10.1177/13524585261429245

**Published:** 2026-03-20

**Authors:** Eva Strijbis, Jop Mostert, Miguel D’Haeseleer, Ester Moral, Luis Brieva Ruiz, Jacynthe Comtois, Joep Killestein, Pavle Repovic, Tarrant McPherson, Gary Cutter, Marcus Koch

**Affiliations:** Amsterdam UMC, Amsterdam, The Netherlands; Department of Neurology, Rijnstate Hospital, Arnhem, The Netherlands; Universitair Ziekenhuis Brussel, Brussels, Belgium; National Multiple Sclerosis Center, Melsbroek, Belgium; Neurology, Hospital Universitario de Bellvitge, L’Hospitalet de Llobregat, Barcelona, Spain; Neuroimmunology Group, Department of Neurology, Hospital Universitari Arnau de Vilanova, Lleida, Spain; Université de Montreal, Montreal, QC, Canada; Amsterdam UMC, Amsterdam, The Netherlands; Swedish Neuroscience Institute Multiple Sclerosis Center, Seattle, WA, USA; Biostatistics and Bioinformatics, Emory University, Atlanta, GA, USA; Department of Biostatistics, School of Public Health, University of Alabama at Birmingham, Birmingham, AL, USA; Clinical Neurosciences, University of Calgary, Calgary, AB, Canada

**Keywords:** Disease modifying therapies, outcome measurement

## Abstract

**Background and Objectives:**

It remains uncertain whether the relative benefit of disease-modifying treatments (DMTs) diminish as patients age because of a natural decline of inflammatory disease activity. To better capture the balance of benefit and harm, the statistical concept of the *Number Needed to Treat* (NNT) provides a useful and easily interpretable metric.

**Methods:**

We examined the relationship between treatment efficacy and age by applying the NNT concept to three pivotal randomized clinical trials of high-efficacy DMTs: AFFIRM, SENTINEL, and DECIDE (3,954 participants together). NNTs were calculated to determine how many individuals within each age group would need to be treated to prevent one additional inflammatory event (by different definitions of significant inflammation).

**Results:**

Inflammatory disease activity decreased with advancing age, resulting in progressively higher NNTs in older participants. For instance, in the SENTINEL trial, the NNT to prevent one additional new/enlarging T2 lesion annually was one for the youngest patients (⩽30 years) compared with 10 for the oldest (>50 years). Similarly, in AFFIRM, the NNT to prevent one relapse was two in the youngest group (⩽30 years) versus four in the oldest (41–50 years).

**Discussion:**

NNTs provide a framework for contextualizing treatment efficacy against potential harms, supporting more individualized therapeutic decision-making.

## Introduction

The risk of developing new clinical relapses and/or focal demyelinating magnetic resonance imaging (MRI) lesions in relapsing-remitting multiple sclerosis (RRMS) decreases with advancing age.^1,2^ This pattern is present throughout the patients’ lifespan. Notably, this association appears to be largely independent of disease duration.^3^

While the immunomodulating effects of disease-modifying treatment (DMT) are not expected to wane with age, the relative benefit that these medications give to patients with RRMS may decline with age, due to the naturally decreasing number of inflammatory events that such drugs aim to prevent. In other words, older patients may remain free of inflammatory events not because they are benefitting from treatment, but as a mere consequence of normal aging. In keeping with this thought, a previous meta-analysis of 38 pivotal DMT trials concluded that the effect of many agents currently used to prevent disability worsening in RRMS disappears from an approximate average age of 53.^4^

Clinical trial results in RRMS are traditionally presented as relative risk reduction or changes in cumulative events for the entire study cohort; for example, by reporting differences in the annualized relapse rate (ARR) between comparative arms. However, relative risk reductions do not inform the reader about the absolute number of events, and, moreover, these results cannot be easily used for clinical counselling of individual patients. The statistical concept of the ‘Number Needed to Treat’ (NNT) offers a measurement of true treatment impact, by estimating the number of patients that need to be treated in order to prevent one additional undesired outcome. In the context of RRMS, the NNT may be the number of patients treated to prevent a single relapse or development of any new MRI lesions. Generally, the NNT is the inverse of absolute risk reduction and, according to the CONSORT statement, should be reported in all clinical trial results.^5^

To investigate the influence of age on the effectiveness of DMT in RRMS, we re-analysed three large trial datasets, all including cohorts of RRMS patients with meaningful inflammatory disease activity at baseline. Our first objective was to describe how the treatment effects of natalizumab and daclizumab may differ in younger compared to older age groups. We calculated NNTs to indicate how many participants in each age group would need to be treated to prevent one additional clinical relapse or new/enlarging T2 MRI lesion per year.^6^

In addition to the NNT, we also examined the effect of treatment on the absence of clinical relapses and new/enlarging T2 lesions, expressed as the percentage of participants with (1) No Evidence of inflammatory Disease Activity (NEIDA)^7^ and (2) Minimal Evidence of inflammatory Disease Activity (MEDA); see below for exact NEIDA and MEDA definitions.^8^ Finally, because especially the ARR and annualized number of new/enlarging T2 lesions (aNewT2) are typically presented as a single average across the entire trial cohort, we added a presentation in deciles of the trial cohorts, to explore the overall distribution of and the impact of extreme values on the average of these outcomes. Our aim in these analyses is not to promote or discourage the use of any specific DMT, such as natalizumab or daclizumab (the latter of which is no longer used in clinical practice), but to explore more informative ways of presenting trial results.

## Methods

### Standard protocol approvals, registrations and patient approvals

Ethical approval for this analysis was granted by the University of Calgary Conjoint Health Research Ethics Board. Ethical approval of the AFFIRM (NCT00027300), and SENTINEL (NCT00030966), and DECIDE (NCT01064401) randomized controlled trials is described in the original publications.^9–11^ All participants gave their written informed consent prior to participation in these clinical trials.

### Trial datasets

AFFIRM and SENTINEL were multicenter phase 3 trials investigating the efficacy of 300 mg natalizumab in patients with RRMS. DECIDE investigated the efficacy and safety of daclizumab 150 mg every 4 weeks in RRMS patients.

In AFFIRM,^9^ enrolment was limited to patients aged 18 to 50 inclusive, with an Expanded Disability Status Scale (EDSS)^12^ score of 0 to 5.0 inclusive, who had experienced at least one medically documented relapse within the 12 months before inclusion. Patients with any DMT exposure in the 6 months before inclusion could not participate. Patients were randomly assigned in a 2:1 ratio to receive either natalizumab or placebo for up to 120 weeks.

SENTINEL^10^ included patients aged 18 to 55 inclusive (EDSS 0 to 5.0) with at least one relapse in the 12 months before inclusion despite interferon (IFN) beta-1a therapy. Patients were randomized in a 1:1 ratio to receive either natalizumab plus IFN beta-1a or IFN beta-1a alone for up to 116 weeks.

DECIDE^11^ included patients with an age of 18 to 55 years and an EDSS score of 0 to 5.0. To be eligible for inclusion, patients had to have either two or more clinical relapses within the previous 3 years, with at least one clinical relapse occurring in the year prior to enrolment, or one or more clinical relapses and at least one new lesion on MRI that was not associated with the clinical relapse within the previous 2 years, with at least one of these events occurring in the 12 months before enrolment. Patients received either daclizumab HYP or IFN beta-1a.

### Outcome measures

In AFFIRM, SENTINEL, and DECIDE, clinical evaluations were scheduled every 12 weeks and included a relapse and EDSS assessment. In AFFIRM and SENTINEL, MRI scans were performed at baseline and then annually, in DECIDE at baseline and week 24, 96, and 144.

For this investigation, we extracted data on demographics, relapses, EDSS scores and MRI scans at baseline. We calculated NNTs to indicate how many participants in each age group would need to be treated to prevent one additional clinical relapse or new/enlarging T2 MRI lesion per year.^6^

We also examined the effect of treatment on the absence of clinical relapses and new/enlarging T2 lesions, expressed as the percentage of participants with (1) No Evidence of inflammatory Disease Activity (NEIDA)^7^ and (2) Minimal Evidence of inflammatory Disease Activity (MEDA). ARR was defined as the number of relapses during follow-up, divided by the number of years of follow-up. The aNewT2 was defined as the number of new/enlarging T2 lesions during follow-up, divided by the number of years of follow-up. NEIDA was defined as the absence of any relapse or new/enlarging T2 lesions during follow-up,^13,14^ and MEDA as the absence of any relapse in combination with fewer than three new/enlarging T2 lesions during follow-up.^8^

### Statistical analysis

We first calculated the numbers of clinical relapses and new/enlarging T2 lesions for the trial populations and the different age groups within each trial. We then calculated absolute risk difference and the percentage risk reduction of ARR and aNewT2 in the active arm relative to the control arm for each age group separately. NNTs were calculated for the active trial arm relative to the control arms of the respective study. We calculated the NNTs needed to prevent one additional relapse or new/enlarging MRI lesion per year using the formula

NNT=100/AbRR

where AbRR is the absolute risk reduction in the active arm in a trial, compared to its control arm.^15^

We then calculated the proportion of participants free of any relapse, with NEIDA and with MEDA for each age group and trial arm separately. Finally, for calculation of the distribution of inflammatory disease activity across the population, we present the trial populations in ARR and aNewT2 deciles. Analyses were done using R, version 4.3.2.^16^

### Data availability

The data used in this study are available upon request from Biogen (Biogen, Cambridge, Massachusetts, USA). Individual participant data collected during the trial was shared after anonymization and on approval of a research proposal and data sharing agreement.

## Results

### Demographics

Baseline characteristics were similar across the three trials. The average age was 36.0 (SD 8.26) years in AFFIRM, 38.9 (SD 7.65) years in SENTINEL and 36.3 (SD 9.34) years in DECIDE. Median EDSS scores at baseline ranged between 2.0 and 2.5 and there were on average 1.5–1.6 relapses in the year prior to enrolment. The average disease duration was slightly higher in SENTINEL (9.2 years, SD 6.53) compared to AFFIRM (7.5 years, SD 6.57) and DECIDE (6.9 years, SD 6.27), while the number of contrast enhancing lesions (CELs) were lower in SENTINEL (0.9, SD 2.2) compared to the other two trials: 2.1 (SD 4.8) in AFFIRM, and 2.1 (SD 5.8) in DECIDE (Table 1). The number of relapses in the year prior to enrolment declined with increasing age at enrolment from 1.62 (SD 1.12) (<30 years) to 1.47 (SD 0.75) (41–50 years) in AFFIRM, 1.58 (SD 0.86) (<30 years) to 1.39 (SD 0.65) (50 years and older) in SENTINEL and 1.65 (SD 0.78) (<30 years) to 1.31 (SD 0.57) (50 years and older) in DECIDE. A similar age-associated decrease was present for the number of CELs at baseline across the three trials (Table 1).

**Table 1.** Baseline characteristics of AFFIRM, SENTINEL, and DECIDE participants by trial arm.

	AFFIRM Placebo	AFFIRM Natalizumab	SENTINEL IFN beta and Placebo	SENTINEL IFN beta and Natalizumab	DECIDE INF beta	DECIDE Daclizumab
n	315	627	582	589	922	919
f/m (n,%)	211/104 (67/33)	449/178 (72/28)	420/162 (72/28)	442/147 (75/25)	627/295 (68/32)	625/294 (68/32)
Age (mean, SD)	36.7, 7.8	35.6, 8.5	39.1, 7.6	38.8, 7.7	36.2, 9.3	36.4, 9.4
Age groups (n, %):						
Up to 30 years	72 (22.9)	196 (31.3)	82 (14.1)	94 (16.0)	280 (30.4)	289 (31.4)
31 to 40 years	128 (40.6)	224 (35.7)	235 (40.4)	238 (40.4)	326 (35.4)	304 (33.1)
41 to 50 years	115 (36.5)	207 (33.0)	235 (40.4)	231 (39.2)	247 (26.8)	258 (28.1)
over 50 years	—	—	30 (5.2)	26 (4.4)	69 (7.5)	68 (7.4)
Disease duration (mean, SD)	7.7, 6.5	7.4, 6.7	9.6, 6.9	8.7, 6.2	6.9, 6.3	7.0, 6.3
EDSS (median, IQR)	2.0, 1.5 to 3.0	2.0, 1.5 to 3.0	2.5, 1.5 to 3.5	2.0, 1.5 to 3.0	2.5, 1.5 to 3.5	2.0, 1.5 to 3.5
Number of relapses in the year before inclusion (mean, SD)	1.5, 0.8	1.5, 0.9	1.5, 0.7	1.5, 0.8	1.6, 0.7	1.5, 0.7
Number of relapses in the year before inclusion by age group (mean, SD):						
Up to 30 years	1.63, 0.88	1.61, 1.20	1.68, 0.89	1.49, 0.84	1.7, 0.80	1.64, 0.76
31 to 40 years	1.48, 0.72	1.50, 0.70	1.44, 0.64	1.49, 0.83	1.59, 0.73	1.51, 0.69
41 to 50 years	1.45, 0.74	1.49, 0.76	1.49, 0.72	1.39, 0.64	1.56, 0.74	1.49, 0.72
over 50 years	—	—	1.4, 0.67	1.39, 0.64	1.36, 0.60	1.27, 0.54
Number of CELs (mean, SD)	2.0, 4.8	2.2, 4.7	0.9, 1.9	0.9, 2.5	2.2, 5.8	2.0, 5.8
Number of CELs at screening by age group (mean, SD):						
Up to 30 years	3.0, 7.0	3.1, 6.1	1.7, 2.7	1.8, 3.2	3.4, 8.3	3.5, 1.9
31 to 40 years	1.7, 3.5	2.3, 4.2	1.0, 2.1	0.8, 2.2	2.3, 5.2	1.5, 2.9
41 to 50 years	1.8, 4.1	1.3, 3.3	0.6, 1.4	0.8, 2.4	1.4, 2.9	1.1, 2.9
over 50 years	—	—	0.2, 0.5	0.3, 0.7	0.5, 1.7	1.0, 1.9

SD, standard deviation; IQR, interquartile range; IFN beta, interferon beta-1a; CELs, contrast enhancing lesions.

### Relapses and relative risk reductions with increasing age

The ARR declined with increasing enrolment age over the course of the natalizumab trials in both control arms, but not in the natalizumab arms (Table 2). In DECIDE, the effect of age was present in both arms but lower in the daclizumab arm.

**Table 2.** Relapse activity during the AFFIRM, SENTINEL and DECIDE trials.

	AFFIRM Placebo	AFFIRM NTZ	SENTINEL IFNβ and Placebo	SENTINEL IFNβ and NTZ	DECIDE IFNβ	DECIDE Daclizumab
n	315	627	582	589	922	919
ARR (mean, SD)	0.64, 0.90	0.21, 0.47	0.70, 0.86	0.28, 0.48	0.39, 0.63	0.23, 0.47
ARR by decile (mean, SD):						
Bottom	—	—	—	—	—	—
Second	—	—	—	—	—	—
Third	—	—	—	—	—	—
Fourth	—	—	0.05, 0.15	—	—	—
Fifth	0.19, 0.23	—	0.45, 0.0	—	—	—
Sixth	0.45, 0.00	—	0.45, 0.0	—	0.09, 0.16	—
Seventh	0.74, 0.22	—	0.78, 0.19	0.35, 0.19	0.40, 0.03	—
Eighth	0.92, 0.06	0.33, 0.20	1.03, 0.20	0.45, 0.0	0.55, 0.08	0.35, 0.13
Ninth	1.40, 0.14	0.46, 0.07	1.60, 0.22	0.63, 0.21	0.91, 0.14	0.57, 0.10
Top	2.77, 1.04	1.28, 0.83	2.67, 0.74	1.42, 0.53	1.93, 0.66	1.38, 0.57
ARR by age group (mean, SD):						
Up to 30 years	0.90, 0.98	0.17, 0.36	0.89, 0.91	0.25, 0.40	0.49, 0.76	0.22, 0.44
31 to 40 years	0.74, 1.05	0.22, 0.60	0.78, 0.86	0.32, 0.56	0.37, 0.59	0.25, 0.52
41 to 50 years	0.49, 0.64	0.22, 0.42	0.60, 0.87	0.26, 0.41	0.35, 0.56	0.24, 0.46
Over 50 years	—	—	0.39, 0.57	0.32, 0.47	0.16, 0.34	0.18, 0.29
NNT to prevent one additional relapse per year compared to control, overall	—	2	—	3	—	7
NNT to prevent one additional relapse per year compared to control, by age group (mean, SD):						
Up to 30 years	—	2	—	2	—	4
31 to 40 years		2		3		9
41 to 50 years		4		3		10
Over 50 years		—		**15**		−50 (NNH)

Table 2 shows the clinical endpoints related to inflammatory disease activity in the treated and control arms of AFFIRM, SENTINEL and DECIDE. The concept of the ‘Number Needed to Treat’ (NNT) offers a measurement of treatment impact, by estimating the number of patients that need to be treated in order to prevent one additional undesired outcome. Endpoints were calculated for the complete trial population as well as the different age groups. NTZ, natalizumab; IFNβ, interferon beta; ARR, annualized relapse rate; SD, standard deviation; NNT, number needed to treat; NNH, number needed to harm.

**Figure 1.**
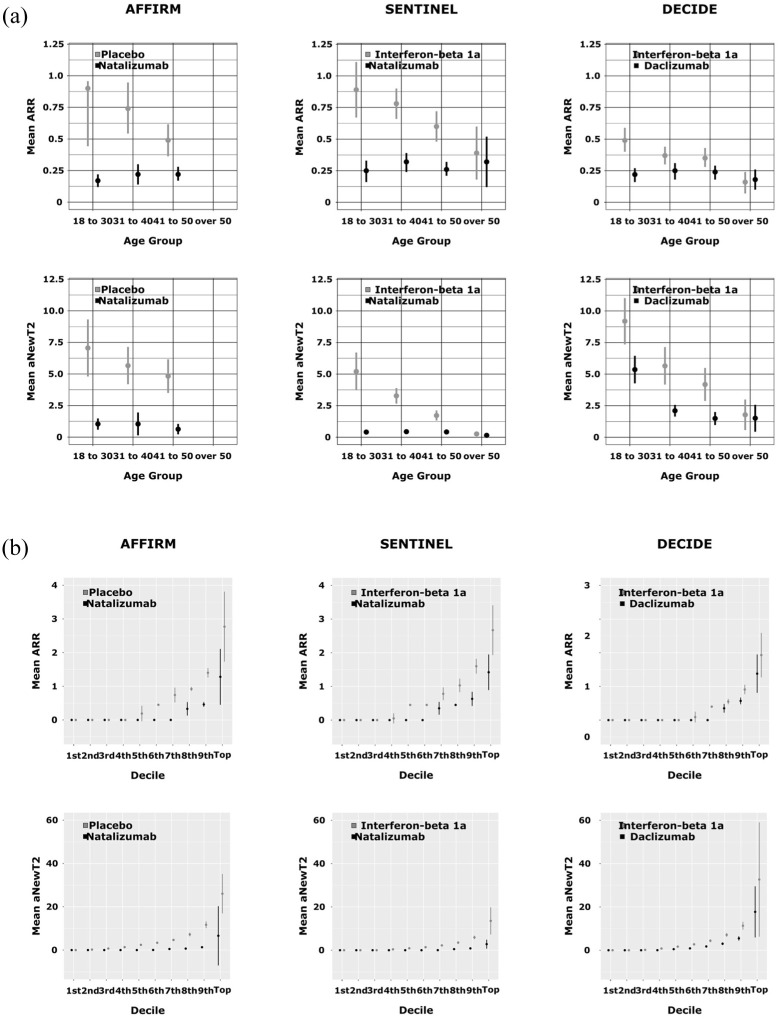
Inflammatory disease activity in trial participants. (a) Mean annualized relapse rate (ARR; ±%95 confidence interval) and mean annualized new T2 lesions (aNewT2; ±SD) per treatment arm in different age groups. The treatment effect, or the difference between the treatment arms, diminishes with advancing age: participants over 50 years of age at baseline benefit meaningfully less from treatment than younger people with RRMS. (b) Mean annualized relapse rate (ARR; ±SD) and mean annualized new T2 lesions (aNewT2; ±SD) in deciles. Disease activity in RRMS trials is unevenly distributed. For example, 40% (first to fourth decile) of even the placebo-treated participants of AFFIRM remained relapse free during follow-up.

When comparing ARR between treatment arms and age groups, we found smaller absolute ARR differences in the older groups (Table 2, Figure 1A). For example: in AFFIRM the ARR was 0.17 (SD 0.36) in the natalizumab arm and 0.90 (SD 0.98) in the placebo arm, leading to a risk difference of 0.73 and a relative risk reduction of 81% in the youngest age group (up to 30 years). In contrast, in the oldest age group (41–50 years) the ARR was 0.22 (SD 0.42) in the natalizumab arm and 0.49 (SD 0.64) in the placebo arm leading to a risk difference of 0.27 and a relative risk reduction of 55%. Similar patterns were present in SENTINEL and DECIDE (Table 2, Figure 1A) although relapses occurred less frequently in the entire trial population in DECIDE.

### MRI activity

Similar to the decline in ARR, there was also a decline in radiological disease activity (Table 3, Figure 1A). For example: in SENTINEL, the youngest age group (up to 30) had a mean aNewT2 of 5.21 (SD 6.59) in the placebo arm, compared to 0.41 (SD 0.86) in the natalizumab arm, which is a relative risk reduction of 92% with an absolute difference of 4.8 new lesions. In the oldest group (50 and older), there were on average 0.26 (SD 0.44) aNewT2 in the placebo arm, compared to 0.15 (SD 0.39) in the natalizumab arm: a reduction of 42% and an absolute difference of 0.11 new/enlarging T2 lesions.

**Table 3.** MRI activity during the AFFIRM, SENTINEL and DECIDE trials.

	AFFIRM Placebo	AFFIRM NTZ	%	SENTINEL IFNβ and Placebo	SENTINEL IFNβ and NTZ	%	DECIDE IFNβ	DECIDE Daclizumab	%
*n*	315	627		582	589		922	919	
aNewT2 (mean, SD)	5.67 (4.70)	0.91 (8.06)		2.77 (4.47)	0.42 (1.07)		6.05 (12.62)	2.92 (6.38)	
aNewT2 by decile (mean, SD):									
Bottom	—	—		—	—		—	—	
Second	0.23 (0.25)	—		—	—		—	—	
Third	0.70 (0.25)	—		—	—		0.18 (0.24)	—	
Fourth	1.35 (0.33)	—		0.40 (0.20)	—		0.79 (0.25)	0.03 (0.12)	
Fifth	2.42 (0.35)	—		0.90 (0.20)	—		1.67 (0.36)	0.50 (0.00)	
Sixth	3.37 (0.37)	0.05 (0.15)		1.40 (0.28)	—		2.73 (0.50)	0.87 (0.24)	
Seventh	4.68 (0.52)	0.50 (0.00)		2.22 (0.25)	0.03 (0.11)		4.40 (0.65)	1.75 (0.25)	
Eighth	7.21 (0.84)	0.68 (0.24)		3.52 (0.57)	0.50 (0.00)		7.10 (0.89)	3.01 (0.60)	
Ninth	11.67 (1.58)	1.31 (0.31)		5.93 (0.81)	0.85 (0.23)		11.32 (1.81)	5.48 (1.03)	
Top	26.03 (9.10)	6.61 (13.67)		13.56 (6.30)	2.81 (2.07)		32.67 (26.41)	17.73 (11.82)	
aNewT2 by age group (mean, SD):									
Up to 30 years	7.06 (9.33)	1.04 (3.09)	85	5.21 (6.59)	0.41 (0.86)	92	9.19 (14.88)	5.36 (9.22)	42
31 to 40 years	5.66 (8.13)	1.04 (6.73)	82	3.28 (4.61)	0.44 (1.26)	86	5.65 (12.91)	2.10 (3.92)	63
41 to 50 years	4.83 (7.03)	0.63 (2.98)	87	1.72 (3.00)	0.42 (0.98)	75	4.18 (9.94)	1.49 (4.10)	64
over 50 years	—	—	—	0.26 (0.44)	0.15 (0.39)	42	1.78 (4.73)	1.51 (4.19)	15
NNT to prevent one additional new or enlarging T2 lesion per year compared to control, overall	—	1		—	1		—	1	
NNT to prevent one additional new or enlarging T2 lesion per year compared to control, by age group (mean, SD):									
Up to 30 years	—	1		—	1		—	1	
31 to 40 years		1			1			1	
41 to 50 years		1			1			1	
over 50 years		—			10			4	

Table 3 shows the MRI endpoints related to inflammatory disease activity in the treated and control arms of AFFIRM, SENTINEL and DECIDE. Endpoints were calculated for the complete trial population as well as the different age groups. NTZ, natalizumab; IFNβ, interferon beta; aNewT2, annualized new T2 lesions; NNT, number needed to treat.

### Numbers needed to treat (NNT) per age group

Tables 2 and 3 show the calculated NNTs for ARR and aNewT2: in SENTINEL, only one patient aged 30 years or younger had to be treated with natalizumab compared to IFN beta-1a to prevent one new/enlarging T2 lesion annually, and 2 patients to prevent one additional relapse. This is in contrast to the oldest age group (SENTINEL, >50 years) where 10 patients had to be treated with natalizumab to prevent one new lesion compared to IFN beta-1a, and 15 patients to prevent 1 new relapse. In AFFIRM, the NNT was 4 to prevent one additional relapse in the oldest group (45–50) and one to prevent one additional new/enlarging T2 lesion. The NNT for the younger age groups was 1 for both relapses and new T2 lesions. In DECIDE, the NNT for relapses could not be calculated since the ARR was higher in the daclizumab arm in the oldest age group compared to the control arm (Table 2).

### Inflammatory disease activity distribution in trial populations

The presentation of ARR and aNewT2 in deciles (Table 2, Table 3, and Figure 1B) shows that the distribution of inflammatory disease activity was uneven in all trial cohorts; 46.5% of participants receiving placebo in AFFIRM, 39.3% of the IFN beta-1a arm of SENTINEL, and 57.8% in the IFN beta-1a arm of DECIDE experienced no relapse during follow-up. This number increased with older age: 49.1% of participants aged 41 to 50 years in the placebo arm in AFFIRM had no relapses, compared to 43.3% in the group aged up to 30 years. Similar patterns were present in the IFN beta-1a arms of SENTINEL and DECIDE, where the percentage of participants with no relapses even increased to 60.0% (SENTINEL) and 76.2% (DECIDE) in the group aged 50 years and older.

As expected, a lower proportion of participants were free from MRI activity in all trials, but we observed a similar effect of age on radiological disease activity: 8.8% of participants up to 30 years in the placebo arm of AFFIRM had no new/enlarging T2 lesions during trial follow-up, compared to 21.8% of participants aged 41 to 50 years. In the IFN beta-1a arm of SENTINEL, 10.4% of participants aged up to 30 years had no MRI activity, while this was 69% in the group of participants 50 years and older. In the IFN beta-1a arm in DECIDE, these numbers were 12.2% in participants aged up to 30, and 50.8% in the group aged over 50 years. NEIDA and MEDA outcomes showed similar patterns, although, interestingly, the percentage of participants aged over 50 with NEIDA in the IFN beta-1a arm in SENTINEL was lower than in the younger 41 to 50 years group (Table 4).

**Table 4.** Proportion of participants without inflammatory disease activity.

	AFFIRM Placebo	AFFIRM NTZ	SENTINEL IFNβ and Placebo	SENTINEL IFNβ and NTZ	DECIDE IFNβ	DECIDE Daclizumab
Participants without relapse activity (%, n)	46.5 (132)	73.0 (428)	39.3 (190)	62.3 (330)	57.8 (477)	71.3 (608)
Participants without relapse activity (%, n):						
Up to 30 years	43.3 (26)	56.7 (34)	30.9 (21)	65.1 (54)	53.0 (133)	73.1 (196)
31 to 40 years	45.7 (53)	54.3 (63)	31.2 (59)	64.0 (137)	57.5 (168)	71.3 (204)
41 to 50 years	49.1 (53)	50.9 (55)	46.7 (92)	59.8 (125)	58.4 (128)	69.5 (166)
over 50 years	—	—	60.0 (18)	58.3 (14)	76.2 (48)	70.0 (42)
Participants without MRI activity (%, n)	15.5 (46)	59.4 (360)	32.2 (176)	69.6 (392)	26.6 (222)	39.5 (343)
Participants without MRI activity (%, n):						
Up to 30 years	8.8 (6)	55.6 (105)	10.4 (8)	66.7 (58)	12.2 (31)	21.7 (60)
31 to 40 years	13.4 (16)	58.1 (125)	25.7 (57)	71.7 (162)	23.5 (69)	41.1 (117)
41 to 50 years	21.8 (24)	64.4 (130)	41.7 (91)	67.0 (150)	40.4 (91)	53.7 (132)
over 50 years	—	—	69.0 (20)	84.6 (22)	50.8 (31)	54.8 (34)
Participants with NEIDA (%, n)	9.5 (28)	43.1 (259)	16.0 (84)	42.2 (231)	17.4 (145)	28.3 (244)
Participants with NEIDA (%, n):						
Up to 30 years	7.4 (5)	42.9 (81)	6.5 (5)	42.4 (36)	7.5 (19)	15.6 (43)
31 to 40 years	6.8 (8)	42.4 (89)	8.1 (17)	45.0 (98)	13.9 (41)	30.5 (87)
41 to 50 years	13.6 (15)	44.3 (89)	23.2 (48)	38.6 (85)	27.4 (61)	37.3 (90)
over 50 years	—	—	16.7 (14)	50.0 (12)	39.3 (24)	39.3 (24)
Participants with MEDA (%, n)	26.0 (76)	68.5 (406)	30.9 (155)	60.6 (321)	34.8 (290)	54.2 (466)
Participants with MEDA (%, n):						
Up to 30 years	16.7 (11)	68.8 (128)	19.7 (14)	63.9 (53)	19.7 (50)	44.5 (122)
31 to 40 years	25.4 (30)	70.2 (146)	20.7 (42)	62.0 (132)	35.6 (105)	55.8 (158)
41 to 50 years	32.4 (35)	66.3 (132)	41.2 (82)	58.1 (122)	43.0 (96)	61.8 (149)
over 50 years	—	—	58.6 (17)	58.3 (14)	63.9 (39)	60.7 (37)

Table 4 shows percentages of participants without relapses, without new or enlarging T2 lesions, without ‘no evidence of inflammatory disease activity (NEIDA)’ and with ‘minimal evidence of disease activity (MEDA) by trial arm, and by age group.

NTZ, natalizumab; IFNβ, interferon beta; %, percent reduction in active arm compared to control arm; aNewT2, annualized new T2 lesions; NNT, number needed to treat.

## Discussion

In this investigation of patient-level data from three large randomized controlled trials in RRMS, we systematically found that the relative benefits of DMT on relapses and MRI activity in RRMS decrease with advancing age. We selected these datasets not to argue about the relative benefits of these specific treatments, but because they represent people with relatively active RRMS. AFFIRM is one of the last trials to include a placebo arm, which makes it especially appropriate for our purposes: this selection of datasets allows us to investigate placebo, IFN beta-1a, natalizumab, and daclizumab.

In our investigation, we found that the benefit of treatment on inflammatory disease activity decreases with advancing age, up to the point where treatment does not offer a substantial benefit over interferon beta-1a in participants over 50 years of age. For example: older participants (especially those over 50 years of age) had a comparable rate of disease activity in DECIDE (for example 76.2% without relapses in the interferon arm, compared to 70.0% in the daclizumab arm. Another example is that 58.6% of participants over the age of 50 had MEDA in the SENTINEL interferon arm versus 58.3% in the natalizumab + interferon arm. The explanation for these results in our opinion is that age has such a powerful effect on inflammatory disease activity, that HET offers few if any benefit over LET in older individuals. Translating these results into NNTs highlights the clinical importance of these findings: in both SENTINEL and DECIDE, we see that the NNT to prevent one additional relapse per year is low in younger participants (e.g. NNT = 2 in SENTINEL and NNT = 4 in DECIDE for the ‘18 to 30 years’ age group) but increases to 15 (SENTINEL) in those over 50; in DECIDE, the group of participants over 50 years even performed worse on treatment, with a theoretical Number Needed to Harm (NNH) of −50 (i.e. one additional relapse occurs per year in one of 50 daclizumab-treated participants, compared to those treated with interferon beta-1a).

Analyses using NNTs work well when comparing two or more interventions that have their impact over the same period of time in similar populations,^17^ features inherent to randomized controlled trials. An important advantage of using the NNT is that it is possible to relate them to NNHs. Even though side effects are notoriously difficult to estimate on trials with a short follow-up, the pattern of a higher NNT in older populations coincides with an expected lower NNH: the risk of hypogammaglobulinemia and serious infections in people with RRMS on DMT, for example, is highest in older patients.^18–20^ The lower risk of inflammatory disease activity in those over 50 was also the rationale for the recent phase 4 DISCOMS non-inferiority trial study, which randomly assigned DMT-treated RRMS patients to stop their treatment.^21^ While this trial could not formally reject the inferiority of no treatment, it showed very little recurrent inflammatory disease activity in either trial arm. Similarly to our study there was a distinction between the impact of age on relapses and MRI lesions but even in the case of new T2 lesions there is an age effect where older individuals develop less inflammatory disease activity. This effect is also underscored but the differences in results between de DISCOMs (average age of 63 years) and DOT-MS (average age of 53 years) trials. This further illustrates the altered risk-benefit ratio of DMT in older patients.

The presentation of ARR and aNewT2 in deciles demonstrates that the distribution of disease activity in RRMS trial cohorts selected for their disease activity (SENTINEL explicitly includes only participants who failed a previous DMT) is unequal which leads to an overestimation of disease activity because of the left-skewed distribution of lesion numbers. Notably, in the placebo arm of AFFIRM, for example, 40% of participants (the lowest four deciles) did not experience a single relapse, despite recent disease activity before enrolment, and despite the fact that they did not receive active treatment. This percentage increases with older age as well. Similarly, although new or enlarging T2 lesions are a much more sensitive marker of RRMS disease activity than relapses, a full 30% of participants in SENTINEL were free from new and/or enlarging T2 lesions throughout the trial. We encourage the addition of our method, or similar variants to present the distribution of outcomes to future trial publications in RRMS.

A limitation of the use of NNTs, as with any efficacy measure, is the difficulty to compare them across different studies, since trial populations tend to change over time. We have witnessed a decrease in ARR and T2 lesion accumulation in newer trial populations over the years perhaps in part as a consequence of increasingly liberal diagnostic criteria.^22,23^ In addition, it was not common practice to include ‘rebaseline’ scans in older trials. This implies that in trials that only have yearly MRI follow-up, radiological treatment effects in the first year are likely underestimated especially in younger people, since a first follow-up scan after treatment initiation may show some additional disease activity attributed to the first period of treatment. A last limitation, is the fact that only few trials to date have included older participants: the upper age limit at inclusion has most often been between 50 and 55 years. This leads to the group of older participants being relatively underrepresented, as well as to smaller groups to analyse. Nevertheless, all three trials showed reduced treatment effects in older patients, or a situation of ‘diminishing returns’: if the goal of DMT is the reduction of all relapses, our analyses suggest that this goal can be achieved with interferon beta-1a as well as with natalizumab or daclizumab in people over 50 years of age. In the development of future trials, higher ages should be included. We also advocate for subgroup analysis of treatment effects in these groups.

By presenting these findings, we hope not to discourage clinicians from using DMT, but rather to refine the discussion of treatment options with aging and otherwise frail patients such as the possibilities of de-escalating or stopping treatment in these age groups. Future treatment trials and research in RRMS should include the often underrepresented older patient groups, especially considering that MS prevalence is now highest in the age group of 55 to 64 years.^24^
